# Supplementary material for: Fresh-Water Mollusks as Biomonitors for Ecotoxicity of Nanomaterials
Source: Nanomaterials (Basel). 2021 Apr 8;11(4):944. doi: 10.3390/nano11040944 (PMC8068117; doi:10.3390/nano11040944)
Supplement: Supplementary file 1 [file nanomaterials-11-00944-s001.pdf]

nanomaterials

MDPI

Fresh-water mollusks as biomonitors for ecotoxicity of nano-materials

Table S1. Characterization of tested NPs samples (TEM data and microdiffraction)

Samples	TEM data	Samples	TEM data
Si_n-$(C_4F_9)_m$	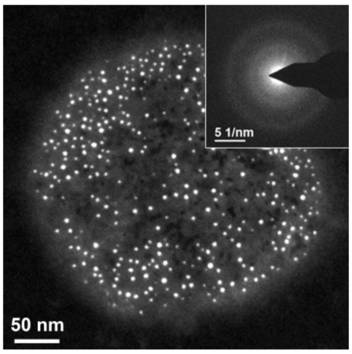	Ag NPs	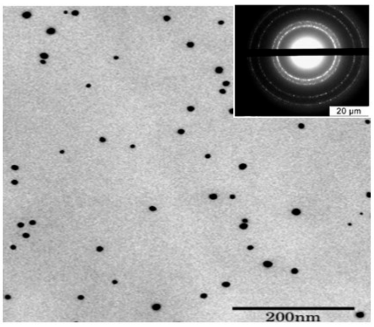
Si_n-$(C_4H_9)_m$	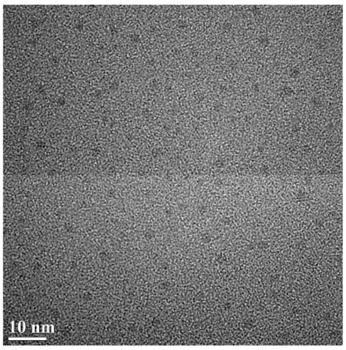	Cu NPs	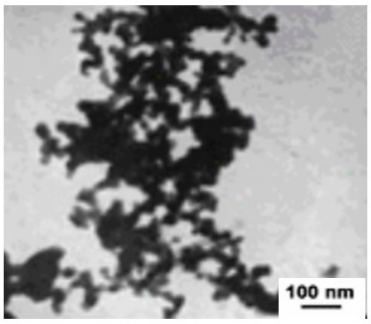
Si_n-$(CH_2)_m$	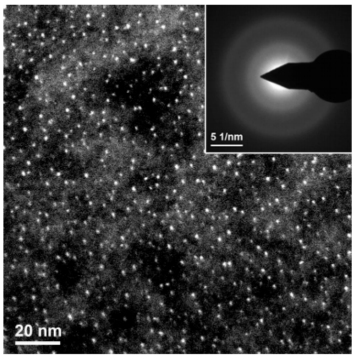	Cu@Ag	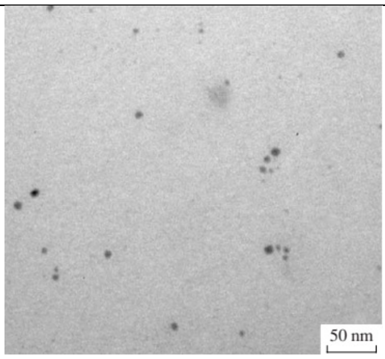
